# Stress events and stress symptoms in Chinese secondary school students: gender and academic year characteristics of the relationship

**DOI:** 10.3389/fpubh.2024.1360907

**Published:** 2024-02-27

**Authors:** Aimin Ma, Shuying Tan, Jin Chen, Hu Lou

**Affiliations:** School of Sports Science, Nantong University, Nantong, China

**Keywords:** secondary school students, stress events, stress symptoms, gender, academic year

## Abstract

**Objective:**

To explore the relationship between stress events and stress symptoms and their gender and academic year characteristics in Chinese secondary school students.

**Methods:**

4,995 secondary school students were investigated by the Adolescent Self-rating Life Events Checklist (ASLEC) and the Calgary Symptoms of Stress Inventory (C-SOSI).

**Results:**

First, there were significant differences in all dimensions and total scores of stress events and stress symptoms between boys and girls in secondary school and between junior high school students and senior high school students. Second, the dimensions and total scores of stress events in secondary school students are positively correlated with the dimensions and total scores of stress symptoms. Third, the influence of punishment on the stress symptoms of secondary school boys is the most obvious, and the influence of punishment, adaption, relationship stress, and learning stress on the stress symptoms of secondary school girls is the most obvious. The influence of punishment on the stress symptoms of junior high school students is the most obvious, and the influence of punishment and relationship on the stress symptoms of senior high school students is the most obvious.

**Conclusion:**

Stress events and stress symptoms of Chinese secondary school students have significant differences in gender and academic year. The same stress event has different influence mechanisms on the stress symptoms of Chinese secondary school students of different genders and different academic years.

## 1 Introduction

Secondary school is not only a period of rapid physical development of adolescents but also a sensitive period of life development. At the same time, they are faced with many growth problems, especially their susceptibility to stressful events in life. Research shows that in the world, secondary school students are the most prone to psychological problems, about 10% of secondary school students have varying degrees of psychological problems (1). And, the psychological problems of secondary school students will not only lead to personal pain and family burden but also bring potential harm to social development. A cross-sectional historical meta-analysis was used to study the group learning stress of 183,165 Junior high school students during the 23 years from 1997 to 2020, and the learning stress of Chinese secondary school students showed a slowly rising trend and has become an important reason for the increasing prominent psychological problems of Chinese secondary school students such as depression, anxiety, and relationship (2).

Since Selye (3) put forward the concept of stress, the impact of stress events on human health as a very important stressor has attracted wide attention. Stress events refer to events that require individuals to use their resources to cope with, but the requirements and demands for individual resources exceed their abilities and cannot be solved, and bring a sense of threat and pressure to them (4). Liu et al. (5) believe that the concept of stress events mainly includes two aspects: one is the intensity, frequency, and duration of stress events, and the other is subjective factors such as subjective cognition, coping style, and social support. The stress events that secondary school students need to face are not the same as those of adults, and their stress events are mainly in study and life, which can be roughly divided into six aspects, namely relationship, learning stress, punishment, loss of relatives and property, health and adaption problems, and other factors (6). For instance, relationship factors include being misunderstood and discriminated against, learning stress factors include failing exams and heavy learning burden, punishment factors include being criticized by teachers and being punished by schools, loss factors include serious illness and death of relatives and friends, health and adaption factors include being away from family and transferring to another school, and other factors include falling in love and fighting. Under the influence of these stressors, individuals may not be able to meet their demands due to insufficient coping ability, which is easy to produce cognitive imbalance and fall into a state of anxiety, which may cause great harm to their physical and mental health. Studies have shown that stress events can cause individual stress responses, leading to various physiological or psychological changes, and even make individuals appear alcohol and drug dependent, Internet addiction, and other situations (7–9). Dong et al. found that adverse events experienced in childhood or adolescence predicted a series of problem behaviors and disease consequences that were harmful to health, and stressful life events increased the risk of emotional and problem behaviors in later development (10, 11). Due to the characteristics of different types of stress events, combined with the occupational nature, environment, and personality of each group of people, their stress responses to stress events are also different. Studies of student populations have found that relationship problems and school-related problems occur more frequently. Adolescent students are more likely than other age groups to experience stress events that are beyond their control and difficult to resolve, while also being more vulnerable to the health effects of stress responses (12). There are also researchers who perceive considerable heterogeneity in individual responses to stress. Open personality moderates the relationship between stress events and depression to a certain extent (13). The more pressure adolescents experience, the more they will use the Internet for emotional management, social compensation, etc. (14). In recent years, China’s basic education reform has been continuously promoted, especially the full implementation of the “Double Reduction” policy. In the field of education in China, the “Double Reduction” policy refers to effectively reducing the excessive homework burden and extracurricular training burden of students in the compulsory education stage. The key measures include: first, reducing the number of extracurricular subject training courses, such as mathematics, Chinese, English, and other subjects; second, increasing the after-school services for students, and most of the after-school services for secondary school students are homework, course learning, and examinations. It remains to be seen whether these changes will alter stress events in secondary school students, and how stress events are related to stress symptoms. Therefore, it is necessary to study the characteristics and changes of secondary school students’ stress events under the new situation.

Stress is a cognitive and behavioral experience process composed of stressors and stress responses. Excessive stress will affect the individual’s physiology, psychology, and behavior, resulting in a variety of stress symptoms, mainly manifested as tension, anxiety, depression, sleep disorders, autonomic nerve dysfunction, negative emotions, and so on (15). In 2022, the National Mental Health Assessment and Development Center of the Institute of Psychology of the Chinese Academy of Sciences surveyed more than 30,000 primary and secondary school students in 29 provinces, autonomous regions, and municipalities across the country, and found that the detection rate of depression among secondary school students was 14.8%, which was lower than the detection rate of 19.0% in 2020, but still higher than the global average depression detection rate of secondary school students (16). Depression, as the most common psychological problem among secondary school students in China, has become an important risk factor for inducing them to commit suicide, and also has a negative impact on their cognitive, social, academic, and other aspects of development (17). Anxiety is an emotional state. Stress and conflict events will lead to individual psychological fluctuations and troubles and fall into an anxiety state. The number of Chinese secondary school students with anxiety symptoms accounts for about 30.0%, which is higher than that of primary school students and college students, and also higher than that of American students at the same period. Moreover, anxiety will also cause significant adverse effects on the physical and mental health and academic performance of secondary school students (18). In addition, studies have found that stress symptoms occur more frequently in Chinese students and there are gender and grade differences (19, 20), but how gender and grade affect stress symptoms in students remains unclear.

Studies have shown that stress events, especially negative life events, are important predictors of stress symptoms such as depression and anxiety (21). Chinese secondary school students are facing the pressure of entering a higher school and employment, and are in a state of competition, tension, and panic for the long term, which is more likely to lead to depression, anxiety, and other problems. The relationship is the direct psychological relationship after communication, and a good relationship is an important factor in maintaining the normal development of individual psychology. Some studies have shown that there is a significant negative correlation between relationship and social anxiety in secondary school students (22). Secondary school students will have a strong sense of loneliness when they feel isolated from others in the process of interpersonal communication. At the same time, they will suffer from depression, loneliness and even bullying in school due to poor adaption, lack of partners, or neglect by peers (23). Due to the vast area of China and uneven regional economic development, many parents in western or rural areas go out to work for years, resulting in a lack of parental care and companionship for left-behind secondary school students, and they are prone to depression, loneliness and other psychological problems (24). Although there have been studies on the relationship between stress events and stress symptoms, it is still not clear what kind of stress events will cause the corresponding stress symptoms. Therefore, this study will clarify the specific manifestations of stress symptoms caused by stress events, and provide a basis for the intervention of psychological stress of secondary school students.

## 2 Methods

### 2.1 Sampling method and survey object

From March to April 2022, in the three economic belts of eastern, western and central China, one city with a high GDP level, one city with a medium GDP level, and one city with a low GPD level (eastern: Nantong, Jiangsu Province, Lishui, Zhejiang Province, and Fuxin, Liaoning Province; western: Chongqing, Yibin, Sichuan Province, and Yili, Xinjiang Uygur Autonomous Region; central: Taiyuan, Shanxi Province, Puyang, Henan Province, and Xiangxi Tujia and Miao Autonomous Prefecture, Hunan Province) will be selected from each economic belt. A junior high school and a senior high school in each of these 9 cities were selected, and a stratified random cluster sampling method was adopted to commission local teachers to distribute 100 paper questionnaires to each grade, totaling 5,400 questionnaires. 5,216 questionnaires were recovered, 221 invalid questionnaires were excluded, and 4,995 valid questionnaires were obtained, with an effective recovery rate of 95.7%.

### 2.2 Survey tools

#### 2.2.1 Adolescent Self-rating Life Events Checklist (ASLEC)

The Adolescent Self-rating Life Events Checklist (ASLEC) revised by Xin Xiuhong et al. (25) was adopted to assess the recent stress events of adolescents. There are 26 items on the scale, including 5 dimensions (relationship stress 4 items, learning stress 4 items, punishment 7 items, loss 6 items, adaption 5 items). Using a six-point scale, a score of “1” means “never occurred,” “3” means “occurred but had little impact,” and “6” means “greatly affected.” The higher the score, the more severely affected the adolescent is by life events. In this study, Cronbach ‘α of this scale was 0.887.

#### 2.2.2 Calgary Symptoms of Stress Inventory (C-SOSI)

The Calgary Symptoms of Stress Inventory (C-SOSI) compiled by Carlson was used for adolescent stress symptoms (26). Two psychology graduate students were invited to translate the questionnaire, and a native English student was invited to translate the Chinese questionnaire back. Then two psychology professors were invited to proofread and revise the Chinese and English questionnaires to form the Chinese version of the stress symptom questionnaire. The questionnaire consists of 56 items, including 8 dimensions (depression 8 items, anger 7 items, sympathetic arousal 9 items, neurological 6 items, muscle tension 8 items, cardiopulmonary 6 items, cognitive disorganization 6 items, upper respiratory 6 items). Using a 5-point scale, a score of “1” means “never” and “5” means “very often,” the higher the score, the more severe the teen’s stress symptoms. In this study, Cronbach ‘α of this scale was 0.935.

### 2.3 Statistical methods

SPSS 25.0 was used to test the reliability and validity of the recovered data, and the frequency, mean and standard deviation of each variable were described and analyzed. Pearson correlation was used to analyze the relationship between stress events and stress symptoms among adolescents, and independent sample T-test was used to analyze the differences between stress events and stress symptoms and their various dimensions in school (junior high school and senior high school) and gender (male and female). Linear regression was used to analyze the influence of different dimensions of stress events on different dimensions of stress symptoms. All the above operations were conducted with “*p* < 0.05” as the standard with statistical significance.

## 3 Results

### 3.1 Demographics

The demographic information of the subjects is shown in Table 1. Among them, 2,486 were boys and 2,509 were girls, and the overall male–female ratio was balanced. In terms of family, there were more non-only children (*N* = 2,953), and the number of resident households was the largest (*N* = 1845), followed by non-agricultural households (*N* = 1,633), and the number of agricultural households (*N* = 1,517) was the smallest. In terms of economic conditions, most of the subjects’ families were in the middle or above level. In terms of academics, most students think their academic performance was in the upper middle level, but there were also cases where their academic performance was not good (*N* = 769), and their academic performance was in the lower middle level (*N* = 974).

**Table 1:** Demographics.

	*N*	%
Gender		
Male	2,486	49.8
Female	2,509	50.2
Academic year		
Junior high school	2,420	48.4
Senior high school	2,575	51.6
Grade		
Grade one of junior high school	826	16.5
Grade two of junior high school	913	18.3
Grade three of junior high school	681	13.6
Grade one of senior high school	876	17.5
Grade two of senior high school	847	17.0
Grade three of senior high school	852	17.1
Whether the only child or not		
The only child	2042	40.9
Non-only child	2,953	59.1
Type of household registration		
Agricultural	1,517	30.4
Non-agricultural	1,633	32.7
Resident	1845	36.9
Economic condition		
Very difficult	582	11.7
Relatively difficult	833	16.7
Intermediate	1,471	29.4
Relatively well off	1,105	22.1
Be well off	1,004	20.1
Bad	769	15.4
Academic performance		
Below the average	974	19.5
Intermediate	1,261	25.2
Above the average	1,064	21.3
Very good	927	18.6
Questionnaire recovery		
Valid	4,995	92.5
Invalid	221	4.1
Lost	184	3.4

### 3.2 The basic situation of stress events and stress symptoms in secondary school students

The descriptive statistical analysis of stress events and symptoms of adolescents was carried out, and the results are shown in Table 2. Among the stress events, the learning stress event had the highest score (1.47 ± 1.179), followed by the relationship stress event (1.32 ± 2.973), adaption event (0.95 ± 0.881), and punishment event (0.90 ± 0.729), and loss event had the lowest score (0.79 ± 0.682). Among the stress symptoms, adolescent depression symptoms (1.26 ± 0.737) were the most prominent, followed by sympathetic arousal symptoms (1.23 ± 0.675), neurological symptoms (1.22 ± 0.650), cognitive disorganization symptoms (1.22 ± 0.644), anger symptoms (1.21 ± 0.678), upper respiratory symptoms (1.21 ± 0.640). Muscle tension symptoms (1.21 ± 0.632) were the least severe.

**Table 2:** Descriptive statistics of all dimensions of stress events and stress symptoms.

Variable	M ± SD	Boy	Girl	Junior high school	Senior high school	t _Gender_	t _Academic year_
Stress event	1.03 ± 0.62	1.00 ± 0.57	1.07 ± 0.67	1.02 ± 0.61	1.05 ± 0.64	−4.025**	−2.093*
Relationship stress	1.32 ± 2.97	1.26 ± 0.89	1.38 ± 1.05	1.29 ± 0.92	1.35 ± 1.02	−4.511**	−2.082*
Learning stress	1.47 ± 1.18	1.40 ± 1.09	1.55 ± 1.26	1.43 ± 1.13	1.51 ± 1.22	−4.604**	−2.304*
Punishment	0.90 ± 0.73	0.89 ± 0.71	0.90 ± 0.75	0.91 ± 0.74	0.89 ± 0.72	−0.613	0.826
Loss	0.79 ± 0.68	0.77 ± 0.66	0.80 ± 0.71	0.79 ± 0.70	0.78 ± 0.67	−1.638	0.568
Adaption	0.95 ± 0.88	0.91 ± 0.80	1.00 ± 0.44	0.89 ± 0.85	1.01 ± 0.89	−3.274**	−4.415**
Stress symptom	1.21 ± 0.55	1.19 ± 0.49	1.23 ± 0.59	1.18 ± 0.49	1.24 ± 0.59	−2.535*	−3.294**
Depression	1.26 ± 0.74	1.24 ± 0.67	1.28 ± 0.80	1.23 ± 0.66	1.28 ± 0.80	−1.832	−2.200*
Anger	1.21 ± 0.68	1.20 ± 0.61	1.22 ± 0.74	1.19 ± 0.62	1.23 ± 0.73	−0.729	−1.821
Sympathetic arousal	1.23 ± 0.68	1.21 ± 0.61	1.28 ± 0.74	1.21 ± 0.61	1.25 ± 0.73	−1.382	−2.093*
Neurological	1.22 ± 0.65	1.20 ± 0.63	1.23 ± 0.67	1.20 ± 0.62	1.23 ± 0.67	−1.502	−1.316
Muscle tension	1.21 ± 0.63	1.19 ± 0.57	1.23 ± 0.69	1.19 ± 0.75	1.23 ± 0.68	−1.973*	−2.082*
Cardiopulmonary arousal	1.20 ± 0.60	1.20 ± 0.59	1.22 ± 0.61	1.20 ± 0.59	1.22 ± 0.61	−1.009	−0.850
Cognitive disorganization	1.22 ± 0.64	1.21 ± 0.58	1.24 ± 0.70	1.20 ± 0.58	1.25 ± 0.70	−1.894	−2.592*
Upper respiratory	1.21 ± 0.64	1.17 ± 0.55	1.24 ± 0.71	1.19 ± 0.56	1.22 ± 0.70	−3.764**	−2.024*

*p<0.05; **p<0.01.

The differences between stress events and stress symptoms were further analyzed in terms of gender and academic year. The results are shown in Table 2. In terms of gender, girls scored significantly higher than boys on stress events and stress symptoms. There were significant differences between male and female students in relationship stress, learning stress, adaption, muscle tension, and upper respiratory symptoms.

In terms of academic year, there are significant differences between junior high school students and senior high school students in stress events and stress symptoms. In the dimensions of relationship stress, learning stress, adaption, depression, sympathetic arousal, muscle tension, cognitive disorganization, and upper respiratory symptoms, the scores of senior high school students were significantly higher than those of junior high school students.

### 3.3 Correlation analysis of stress events and stress symptoms in secondary school students

The correlation analysis of adolescent stress events and stress symptoms was conducted. The results are shown in Table 3. There was a significant positive correlation between stress life events and stress symptoms in adolescents (*r* = 0.553, *p* < 0.01), and all dimensions of stressful life events were significantly positively correlated with all dimensions of stress symptoms, and the correlation remained valid after gender and academic year were divided (see Tables 4–7), and the relationship between the variables met the subsequent hypothesis.

**Table 3:** Correlation analysis of all dimensions of stress events and stress symptoms.

Variable	Stress event	Relationship stress	Learning stress	Punishment	Loss	Adaption
Stress symptom	0.553**	0.413**	0.395**	0.454**	0.401**	0.312**
Depression	0.481**	0.345**	0.338**	0.415**	0.345**	0.269**
Anger	0.504**	0.338**	0.353**	0.427**	0.376**	0.296**
Sympathetic arousal	0.500**	0.369**	0.349**	0.406**	0.373**	0.288**
Neurological	0.435**	0.319**	0.298**	0.375**	0.316**	0.243**
Muscle tension	0.472**	0.349**	0.347**	0.393**	0.333**	0.261**
Cardiopulmonary arousal	0.478**	0.357**	0.323*	0.414**	0.361**	0.253**
Cognitive disorganization	0.501**	0.368**	0.338**	0.433**	0.365**	0.281**
Upper respiratory	0.527**	0.396**	0.362**	0.427**	0.383**	0.312**

*p<0.05; **p<0.01.

**Table 4:** Correlation analysis of all dimensions of stress events and stress symptoms in male students.

Variable	Stress event	Relationship stress	Learning stress	Punishment	Loss	Adaption
Stress symptom	0.481**	0.353**	0.333**	0.432**	0.373**	0.171**
Depression	0.419**	0.303**	0.275**	0.396**	0.327**	0.145**
Anger	0.441**	0.290**	0.300**	0.421**	0.334**	0.167**
Sympathetic arousal	0.426**	0.306**	0.289**	0.377**	0.334**	0.166**
Neurological	0.401**	0.285**	0.248**	0.378**	0.316**	0.156**
Muscle tension	0.443**	0.301**	0.300**	0.422**	0.338**	0.163**
Cardiopulmonary arousal	0.456**	0.327**	0.282**	0.422**	0.355**	0.185**
Cognitive disorganization	0.487**	0.342**	0.303**	0.458**	0.375**	0.197**
Upper respiratory	0.510**	0.377**	0.312**	0.459**	0.397**	0.217**

**p<0.01.

**Table 5:** Correlation analysis of all dimensions of stress events and stress symptoms in female students.

Variable	Stress event	Relationship stress	Learning stress	Punishment	Loss	Adaption
Stress symptom	0.603**	0.453**	0.436**	0.473**	0.423**	0.412**
Depression	0.523**	0.373**	0.381**	0.432**	0.359**	0.358**
Anger	0.547**	0.371**	0.390**	0.434**	0.409**	0.388**
Sympathetic arousal	0.550**	0.411**	0.390**	0.431**	0.404**	0.373**
Neurological	0.462**	0.345**	0.338**	0.371**	0.316**	0.314**
Muscle tension	0.491**	0.382**	0.379**	0.371**	0.328**	0.331**
Cardiopulmonary arousal	0.498**	0.382**	0.357**	0.406**	0.366**	0.311**
Cognitive disorganization	0.510**	0.384**	0.361**	0.415**	0.358**	0.339**
Upper respiratory	0.536**	0.406**	0.393**	0.408**	0.374**	0.375**

**p<0.01.

**Table 6:** Correlation analysis of all dimensions of stress events and stress symptoms in junior high school students.

Variable	Stress event	Relationship stress	Learning stress	Punishment	Loss	Adaption
Stress symptom	0.486**	0.363**	0.355**	0.433**	0.380**	0.212**
Depression	0.423**	0.309**	0.302**	0.398**	0.316**	0.187**
Anger	0.521**	0.338**	0.349**	0.486**	0.422**	0.263**
Sympathetic arousal	0.499**	0.346**	0.338**	0.448**	0.404**	0.251**
Neurological	0.396**	0.280**	0.268**	0.373**	0.310**	0.182**
Muscle tension	0.436**	0.304**	0.320**	0.408**	0.331**	0.192**
Cardiopulmonary arousal	0.455**	0.332**	0.313**	0.415**	0.359**	0.211**
Cognitive disorganization	0.489**	0.347**	0.328**	0.454**	0.379**	0.242**
Upper respiratory	0.518**	0.374**	0.360**	0.467**	0.403**	0.253**

**p<0.01.

**Table 7:** Correlation analysis of all dimensions of stress events and stress symptoms in senior high school students.

Variable	Stress event	Relationship stress	Learning stress	Punishment	Loss	Adaption
Stress symptom	0.605**	0.448**	0.423**	0.478**	0.424**	0.371**
Depression	0.525**	0.370**	0.363**	0.435**	0.373**	0.317**
Anger	0.492**	0.338**	0.356**	0.382**	0.342**	0.317**
Sympathetic arousal	0.502**	0.385**	0.358**	0.376**	0.352**	0.310**
Neurological	0.468**	0.348**	0.322**	0.378**	0.323**	0.285**
Muscle tension	0.500**	0.381**	0.366**	0.385**	0.338**	0.302**
Cardiopulmonary arousal	0.498**	0.378**	0.330**	0.413**	0.364**	0.286**
Cognitive disorganization	0.511**	0.382**	0.345**	0.423**	0.361**	0.302**
Upper respiratory	0.536**	0.412**	0.364**	0.403**	0.374**	0.346**

**p<0.01.

### 3.4 Regression analysis of stress symptoms on stress events in secondary school students

In order to further understand the relationship between stress events and stress symptoms, a linear regression method was adopted, with each dimension of stress events as independent variables and each dimension of stress symptoms as dependent variables. The results are shown in Tables 8–11. For male students, punishment events (*β* = 0.248, *p*<0.01) had the greatest influence on depression symptoms, followed by relationship stress events (*β* = 0.144, *p*<0.01), loss events (*β* = 0.109, *p*<0.01) and learning stress events (*β* = 0.108, *p*<0.01). In terms of anger symptoms, punishment (*β* = 0.272, *p*<0.01) was the most influential event, followed by learning stress (*β* = 0.126, *p*<0.01), relationship stress (*β* = 0.106, *p*<0.01) and loss event (*β* = 0.084, *p*<0.01). In terms of sympathetic arousal symptoms, the most influential events were punishment events (*β* = 0.193, *p*<0.01), followed by relationship stress events (*β* = 0.141, *p*<0.01), learning stress events (*β* = 0.126, *p*<0.01), and loss events (*β* = 0.113, *p*<0.01). In terms of neurological symptoms, punishment events (*β* = 0.232, *p*<0.01) had the greatest influence, followed by relationship stress events (*β* = 0.138, *p*<0.01), loss events (*β* = 0.104, *p*<0.01) and learning stress events (*β* = 0.084, *p*<0.01). In terms of muscle tension symptoms, the most influential event was punishment (*β* = 0.267, *p*<0.01), followed by learning stress (*β* = 0.134，*p*<0.01), relationship stress (*β* = 0.121, *p*<0.01) and loss (*β* = 0.098, *p*<0.01). In terms of cardiopulmonary symptoms, the most influential event was still punishment events (*β* = 0.254, *p*<0.01), the second was relationship stress events (*β* = 0.163, *p*<0.01), followed by loss events (*β* = 0.115, *p*<0.01) and learning stress events (*β* = 0.097, *p*<0.01). In terms of cognitive disorganization symptoms, punishment events (*β* = 0.286, *p*<0.01) had the greatest impact, followed by relationship stress events (*β* = 0.151, *p*<0.01), loss events (*β* = 0.112, *p*<0.01), learning stress events (*β* = 0.108, *p*<0.01) and adaption events (*β* = 0.032, *p*<0.01). In terms of upper respiratory symptoms, punishment events (*β* = 0.258, *p*<0.01) had the greatest impact, followed by relationship stress events (*β* = 0.195, *p*<0.01), loss events (*β* = 0.125, *p*<0.01), learning stress events (*β* = 0.106, *p*<0.01) and adaption events (*β* = 0.053, *p*<0.01).

**Table 8:** Regression effect analysis (Male).

Variable	*β*	se	*t*
Dependent variable: depression			
Relationship stress	0.144	0.033	6.682**
Learning stress	0.108	0.027	5.071**
Punishment	0.248	0.028	9.705**
Loss	0.109	0.034	4.353**
Adaption	0.001	0.025	0.031
Dependent variable: Anger			
Relationship stress	0.106	0.026	4.919**
Learning stress	0.126	0.021	5.898**
Punishment	0.272	0.022	10.626**
Loss	0.084	0.028	3.350**
Adaption	0.020	0.020	0.972
Dependent variable: sympathetic arousal			
Relationship stress	0.141	0.034	6.501**
Learning stress	0.126	0.027	5.842**
Punishment	0.193	0.028	7.466**
Loss	0.113	0.035	4.458**
Adaption	0.039	0.026	1.819
Dependent variable: neurological			
Relationship stress	0.138	0.023	6.291**
Learning stress	0.084	0.019	3.860**
Punishment	0.232	0.020	8.928**
Loss	0.104	0.024	4.081**
Adaption	0.018	0.018	0.833
Dependent variable: muscle tension			
Relationship stress	0.121	0.028	5.659**
Learning stress	0.134	0.023	6.349**
Punishment	0.267	0.023	10.532**
Loss	0.098	0.029	3.963**
Adaption	0.010	0.022	0.488
Dependent variable: cardiopulmonary arousal			
Relationship stress	0.163	0.021	7.695**
Learning stress	0.097	0.017	4.598**
Punishment	0.254	0.018	10.070**
Loss	0.115	0.022	4.653**
Adaption	0.028	0.017	1.369
Dependent variable: cognitive disorganization			
Relationship stress	0.151	0.021	7.268**
Learning stress	0.108	0.017	5.216**
Punishment	0.286	0.017	11.560**
Loss	0.112	0.022	4.616**
Adaption	0.032	0.016	1.589**
Dependent variable: upper respiratory			
Relationship stress	0.195	0.019	9.548**
Learning stress	0.106	0.016	5.233**
Punishment	0.258	0.016	10.612**
Loss	0.125	0.020	5.277**
Adaption	0.053	0.015	2.672**

**p<0.01.

**Table 9:** Regression effect analysis (Female).

Variable	*β*	se	*t*
Dependent variable: depression			
Relationship stress	0.154	0.032	7.315**
Learning stress	0.156	0.027	7.452**
Punishment	0.247	0.031	9.967**
Loss	0.009	0.038	0.359
Adaption	0.170	0.026	8.246**
Dependent variable: anger			
Relationship stress	0.115	0.026	5.515**
Learning stress	0.162	0.022	7.804**
Punishment	0.194	0.025	7.901**
Loss	0.090	0.031	3.74**
Adaption	0.196	0.021	9.539**
Dependent variable: sympathetic arousal			
Relationship stress	0.179	0.033	8.583**
Learning stress	0.154	0.027	7.415**
Punishment	0.174	0.032	7.083**
Loss	0.089	0.039	3.677**
Adaption	0.167	0.027	8.132**
Dependent variable: neurological			
Relationship stress	0.160	0.021	7.231**
Learning stress	0.129	0.017	5.915**
Punishment	0.191	0.020	7.363**
Loss	0.026	0.025	1.023
Adaption	0.143	0.017	6.631**
Dependent variable: muscle tension			
Relationship stress	0.196	0.028	9.084**
Learning stress	0.189	0.023	8.812**
Punishment	0.153	0.027	6.027**
Loss	0.021	0.033	0.824
Adaption	0.141	0.023	6.657**
Dependent variable: cardiopulmonary arousal			
Relationship stress	0.176	0.019	8.131**
Learning stress	0.132	0.016	6.185**
Punishment	0.198	0.018	7.788**
Loss	0.078	0.022	3.106**
Adaption	0.107	0.015	5.078**
Dependent variable: cognitive disorganization			
Relationship stress	0.180	0.021	8.395**
Learning stress	0.135	0.017	6.351**
Punishment	0.193	0.020	7.628**
Loss	0.058	0.025	2.331*
Adaption	0.139	0.017	6.610**
Dependent variable: upper respiratory			
Relationship stress	0.186	0.021	8.939**
Learning stress	0.191	0.018	9.222**
Punishment	0.145	0.020	5.938**
Loss	0.061	0.025	2.515*
Adaption	0.190	0.017	9.289**

*p<0.05; **p<0.01.

**Table 10:** Regression effect analysis (Junior high school student).

Variable	*β*	se	*t*
Dependent variable: depression			
Relationship stress	0.134	0.031	6.149**
Learning stress	0.127	0.025	5.870**
Punishment	0.265	0.028	9.771**
Loss	0.054	0.034	2.025*
Adaption	0.020	0.029	0.909
Dependent variable: anger			
Relationship stress	0.097	0.025	4.645**
Learning stress	0.133	0.020	6.432**
Punishment	0.292	0.022	11.305**
Loss	0.123	0.027	4.858**
Adaption	0.049	0.023	2.305*
dependent variable: sympathetic arousal			
Relationship stress	0.133	0.032	6.264**
Learning stress	0.131	0.025	6.220**
Punishment	0.226	0.028	8.560**
Loss	0.133	0.035	5.127**
Adaption	0.060	0.029	2.764**
Dependent variable: neurological			
Relationship stress	0.107	0.022	4.805**
Learning stress	0.097	0.018	4.401**
Punishment	0.244	0.020	8.821**
Loss	0.078	0.025	2.886**
Adaption	0.021	0.020	0.945
Dependent variable: muscle tension			
Relationship stress	0.108	0.027	4.975**
Learning stress	0.152	0.022	7.050**
Punishment	0.262	0.024	9.682**
Loss	0.065	0.030	2.461*
Adaption	0.022	0.025	0.977
Dependent variable: cardiopulmonary arousal			
Relationship stress	0.142	0.021	6.607**
Learning stress	0.122	0.017	5.692**
Punishment	0.240	0.018	8.979**
Loss	0.107	0.023	4.085**
Adaption	0.024	0.019	1.091
Dependent variable: cognitive disorganization			
Relationship stress	0.135	0.020	6.401**
Learning stress	0.123	0.016	5.874**
Punishment	0.278	0.018	10.589**
Loss	0.097	0.022	3.771**
Adaption	0.043	0.018	2.004*
Dependent variable: upper respiratory			
Relationship stress	0.166	0.019	8.010**
Learning stress	0.150	0.015	7.323**
Punishment	0.257	0.017	9.985**
Loss	0.101	0.021	4.009**
Adaption	0.051	0.017	2.440*

*p<0.05; **p<0.01.

**Table 11:** Regression effect analysis (Senior high school student).

Variable	*β*	se	*t*
Dependent variable: depression			
Relationship stress	0.160	0.033	7.704**
Learning stress	0.146	0.028	7.098**
Punishment	0.245	0.031	10.476**
Loss	0.070	0.038	3.024**
Adaption	0.134	0.024	6.767**
Dependent variable: anger			
Relationship stress	0.131	0.028	6.064**
Learning stress	0.167	0.023	7.830**
Punishment	0.179	0.025	7.398**
Loss	0.062	0.031	2.571*
Adaption	0.163	0.020	7.942**
Dependent variable: sympathetic arousal			
Relationship stress	0.191	0.035	8.961**
Learning stress	0.158	0.029	7.476**
Punishment	0.150	0.032	6.233**
Loss	0.079	0.040	3.329**
Adaption	0.141	0.025	6.946**
Dependent variable: neurological			
Relationship stress	0.184	0.022	8.481**
Learning stress	0.118	0.018	5.482**
Punishment	0.192	0.020	7.868**
Loss	0.060	0.025	2.459*
Adaption	0.119	0.015	5.778**
Dependent variable: Muscle tension			
Relationship stress	0.205	0.029	9.649**
Learning stress	0.176	0.024	8.366**
Punishment	0.171	0.027	7.171**
Loss	0.058	0.033	2.439*
Adaption	0.114	0.021	5.637**
Dependent variable: cardiopulmonary arousal			
Relationship stress	0.193	0.019	9.091**
Learning stress	0.107	0.016	5.076**
Punishment	0.219	0.018	9.155**
Loss	0.091	0.022	3.820**
Adaption	0.097	0.014	4.777**
Dependent variable: cognitive disorganization			
Relationship stress	0.192	0.022	9.064**
Learning stress	0.123	0.018	5.850**
Punishment	0.212	0.020	8.862**
Loss	0.079	0.025	3.338**
Adaption	0.113	0.016	5.616**
Dependent variable: upper respiratory			
Relationship stress	0.211	0.021	10.233**
Learning stress	0.163	0.018	7.971**
Punishment	0.155	0.020	6.705**
Loss	0.087	0.025	3.793**
Adaption	0.176	0.015	8.962**

*p<0.05; **p<0.01.

For female students, punishment events (*β* = 0.247, *p* < 0.01) had the greatest influence on depression symptoms, followed by adaption events (*β* = 0.170, *p* < 0.01), learning stress events (*β* = 0.156, *p* < 0.01), and relationship stress events (*β* = 0.154, *p* < 0.01). In terms of anger symptoms, the most influential event was adaption (*β* = 0.196, *p* < 0.01), followed by punishment (*β* = 0.194, *p* < 0.01), learning stress (*β* = 0.162, *p* < 0.01), relationship stress (*β* = 0.115, *p* < 0.01) and loss (*β* = 0.090, *p* < 0.01). In terms of sympathetic arousal symptoms, the most influential event was relationship stress (*β* = 0.179, *p* < 0.01), followed by punishment (*β* = 0.174, *p* < 0.01), adaption (*β* = 0.167, *p* < 0.01), learning stress (*β* = 0.154, *p* < 0.01) and loss (*β* = 0.089, *p* < 0.01). In terms of neurological symptoms, punishment events (*β* = 0.191, *p* < 0.01) had the greatest influence, followed by relationship stress events (*β* = 0.160, *p* < 0.01), adaption events (*β* = 0.143, *p* < 0.01), and learning stress events (*β* = 0.129, *p* < 0.01). In terms of muscle tension symptoms, the most influential event was relationship stress (*β* = 0.196, *p* < 0.01), followed by learning stress (*β* = 0.189, *p* < 0.01), punishment (*β* = 0.153, *p* < 0.01) and adaption (*β* = 0.141, *p* < 0.01). In terms of cardiopulmonary symptoms, the most influential event was punishment (*β* = 0.198, *p* < 0.01), followed by relationship stress (*β* = 0.176, *p* < 0.01), learning stress (*β* = 0.132, *p* < 0.01), adaption (*β* = 0.107, *p* < 0.01). *p* < 0.01) and loss (*β* = 0.078, *p* < 0.01). In terms of cognitive disorganization symptoms, punishment events (*β* = 0.193, *p* < 0.01) had the greatest impact, followed by relationship stress events (*β* = 0.180, *p* < 0.01), adaption events (*β* = 0.139, *p* < 0.01), learning stress events (*β* = 0.135, *p* < 0.01) and loss events (*β* = 0.058, *p* < 0.05). In terms of upper respiratory symptoms, learning stress events (*β* = 0.191, *p* < 0.01) had the greatest impact, followed by adaption events (β = 0.190, *p* < 0.01), relationship stress events (*β* = 0.186, *p* < 0.01), punishment events (*β* = 0.145, *p* < 0.01) and loss events (*β* = 0.061, *p* < 0.05).

For junior high school students, punishment events had the greatest impact on depression symptoms (*β* = 0.265, *p* < 0.01), followed by relationship stress events (*β* = 0.134, *p* < 0.01), learning stress events (*β* = 0.127, *p* < 0.01) and loss events (*β* = 0.054, *p* < 0.05). In terms of anger symptoms, the most influential event was punishment (*β* = 0.292, *p* < 0.01), followed by learning stress (*β* = 0.133, *p* < 0.01), loss (*β* = 0.123, *p* < 0.01), relationship stress (*β* = 0.097, *p* < 0.01) and adaption (*β* = 0.049, *p* < 0.05). In terms of sympathetic arousal symptoms, the most influential event was punishment (*β* = 0.226, *p* < 0.01), followed by relationship stress (*β* = 0.133, *p* < 0.01), loss (*β* = 0.133, *p* < 0.01), learning stress (*β* = 0.131, *p* < 0.01) and adaption (*β* = 0.060, *p* < 0.01). In terms of neurological symptoms, punishment events had the greatest influence (*β* = 0.244, *p* < 0.01), followed by relationship stress events (*β* = 0.107, *p* < 0.01), learning stress events (*β* = 0.097, *p* < 0.01) and loss events (*β* = 0.078, *p* < 0.01). In terms of muscle tension symptoms, the most influential event was punishment (*β* = 0.262, *p* < 0.01), followed by learning stress (*β* = 0.152, *p* < 0.01), relationship stress (*β* = 0.108, *p* < 0.01) and loss (*β* = 0.065, *p* < 0.05). In terms of cardiopulmonary symptoms, the most influential events were punishment (*β* = 0.240, *p* < 0.01), followed by relationship stress events (*β* = 0.142, *p* < 0.01), learning stress events (*β* = 0.122, *p* < 0.01) and loss events (*β* = 0.107, *p* < 0.01). In terms of cognitive disorganization symptoms, punishment events had the greatest impact (*β* = 0.278, *p* < 0.01), followed by relationship stress events (*β* = 0.135, *p* < 0.01), learning stress events (*β* = 0.123, *p* < 0.01), loss events (*β* = 0.097, *p* < 0.01) and adaption events (*β* = 0.043, *p* < 0.05). In terms of upper respiratory symptoms, punishment events had the greatest impact (*β* = 0.257, *p* < 0.01), followed by relationship stress events (*β* = 0.166, *p* < 0.01), learning stress events (*β* = 0.150, *p* < 0.01), loss events (*β* = 0.101, *p* < 0.01) and adaption events (*β* = 0.051, *p* < 0.05).

For senior high school students, punishment events had the greatest impact on depression symptoms (*β* = 0.245, *p* < 0.01), followed by relationship stress events (*β* = 0.160, *p* < 0.01), learning stress events (*β* = 0.146, *p* < 0.01), adaption events (*β* = 0.134, *p* < 0.01) and loss events (*β* = 0.070, *p* < 0.01). In terms of anger symptoms, the most influential event was punishment (*β* = 0.179, *p* < 0.01), followed by learning stress (*β* = 0.167, *p* < 0.01), adaption (*β* = 0.163, *p* < 0.01), relationship stress (*β* = 0.131, *p* < 0.01) and loss (*β* = 0.062, *p* < 0.05). In terms of sympathetic arousal symptoms, relationship stress (*β* = 0.191, *p* < 0.01) was the most influential event, followed by learning stress (*β* = 0.158, *p* < 0.01), punishment (*β* = 0.150, *p* < 0.01), adaption (*β* = 0.141, *p* < 0.01) and loss (*β* = 0.079, *p* < 0.01). In terms of neurological symptoms, punishment events had the greatest influence (*β* = 0.192, *p* < 0.01), followed by relationship stress events (*β* = 0.184, *p* < 0.01), adaption events (*β* = 0.119, *p* < 0.01), learning stress events (*β* = 0.118, *p* < 0.01) and loss events (*β* = 0.060, *p* < 0.05). In terms of muscle tension symptoms, the most influential event was relationship stress (*β* = 0.205, *p* < 0.01), followed by learning stress (*β* = 0.176, *p* < 0.01), punishment (*β* = 0.171, *p* < 0.01), adaption (*β* = 0.114, *p* < 0.01) and loss (*β* = 0.058, *p* < 0.05). In terms of cardiopulmonary symptoms, the most influential event was punishment (*β* = 0.219, *p* < 0.01), followed by relationship stress (*β* = 0.193, *p* < 0.01), learning stress (*β* = 0.107, *p* < 0.01), adaption (*β* = 0.097, *p* < 0.01), (*p* < 0.01) and loss (*β* = 0.091, *p* < 0.01). In terms of cognitive disorganization symptoms, punishment events had the greatest impact (*β* = 0.212, *p* < 0.01), followed by relationship stress events (*β* = 0.192, *p* < 0.01), learning stress events (*β* = 0.123, *p* < 0.01), adaption events (*β* = 0.113, *p* < 0.01) and loss (*β* = 0.079, *p* < 0.01). In terms of upper respiratory symptoms, relationship stress events had the greatest impact (*β* = 0.211, *p* < 0.01), followed by adaption events (*β* = 0.176, *p* < 0.01), learning stress events (*β* = 0.163, *p* < 0.01), punishment (*β* = 0.155, *p* < 0.01) and loss (*β* = 0.087, *p* < 0.01).

## 4 Discussion

### 4.1 Descriptive statistical analysis of stress events in secondary school students

The results of this study show that learning stress, relationship stress and adaption problems of Chinese secondary school students are obvious, which is consistent with the results of previous studies (27–29). First of all, there are many sources of secondary school students’ learning stress, and are more complicated. Their learning stress not only comes from the stress of high school entrance examinations and college entrance examinations but also the stress from parents’ expectations and competition between classmates. In addition, the stress of academic requirement improvement and the stress of students’ self-development are also important reasons for the abnormal increase in secondary school students’ learning stress. Secondly, the relationship stress of secondary school students is also obvious, which is mainly manifested in two aspects: parent relationships and peer relationships. Chinese parents generally pay more attention to their children’s academic performance and expect their children to succeed (30). Moreover, the generation gap between parents and children and the rebellious psychology of secondary school students make the relationship between them and their parents more tense. At the same time, secondary school students in adolescence begin to have their own social circle and gradually pay attention to their relationship relationships with peers, but the relationship communication of secondary school students in real life often appears shy, indifferent, and distant. What’s more, there are still some bad phenomena such as school bullying and violence, which make secondary school students’ relationship stress and school adaption problems become increasingly serious in some areas.

There are significant gender differences in stress events of secondary school students, which is basically consistent with previous studies (31, 32). The total score of stress events and the scores of relationship stress, learning stress, and adaption of female students were significantly higher than those of male students, indicating that female students felt more stress than male students. Perhaps due to the difference in physical conditions, girls are worse than boys in terms of physical strength and energy, which makes girls need to pay more to achieve the same goal under the same conditions. Meanwhile, girls are more sentimental than boys in character and girls are more competitive, study hard, and psychological stress is obviously higher than boys.

Stress events of junior high school students are significantly different in different academic years, which is basically consistent with previous studies (33, 34). The total score of stress events, and the scores of relationship stress, learning stress, and adaption of senior high school students are significantly higher than those of junior high school students. First, from the perspective of entering a higher school, senior high school students have higher stress than junior high school students, especially in psychological stress. Since junior high school students are mostly not self-conscious enough and still need parental supervision, the greater the pressure will be, while senior high school students are more mature and know what they want. Second, from the perspective of learning tasks, the knowledge and the difficulty of senior high school courses are far more than that of junior high school courses. Third, from the perspective of learning methods, junior high school students have relatively simple learning methods and are accustomed to relying on teachers, while senior high school students put more emphasis on students’ independent learning and independent problem-solving ability. Fourth, senior high school students’ reading, writing, calculation, understanding, analysis, and other requirements have been greatly improved. In addition, adolescents are at the peak of relationship communication, they need to face the stress from classmates, teachers, parents, and other aspects, while the range of communication and relationships of senior high school students are complex, especially in adolescence. Senior high school students are more impulsive and rebellious than junior high school students, and they start to pay attention to the opposite sex. However, due to the influence of traditional ideas and family education, senior high school students are prone to self-repression and can not effectively deal with interpersonal relations.

### 4.2 Descriptive statistical analysis of stress symptoms in secondary school students

The results of this study show that the symptoms of depression, sympathetic arousal, neurological, and cognitive disorganization in Chinese secondary school students are relatively obvious, which is basically consistent with the results of previous studies (22, 35, 36). Firstly, the depression symptoms in secondary school students are not depression in the clinical sense, but a kind of negative emotional experience characterized by persistent low mood. When they are in an emotional state of depression will not feel happy, lack interest, often produce a sense of powerlessness, despair, easy to cry, weakened social function, etc., mainly in the emotional, physiological, thinking, and behavioral aspects, such as emotional will be upset for no reason, feeling heavy in severe cases, sad, despair for the future, etc. Physiological will appear appetite loss or overeating lead to significant weight changes. Besides, easy to wake up early or dream more, insomnia or sleep cannot wake up, and the body is often in inexplicable pain, but no organic disease. Thinking will cause memory decline, difficulty in concentration, reduced learning efficiency, and grades decline. Behavioral performance is lack of interest, always feeling tired, staying in bed in severe cases, not wanting to go to school, self-isolation, a tendency to silence and solitude, easy to impulsive, extreme behavior, and even suicidal, self-harm thoughts or behaviors. Secondly, the symptoms of sympathetic arousal in secondary school students mainly include increased sweating, rapid heartbeat, gastrointestinal discomfort, etc., and other symptoms such as sleep disorders may also occur. Finally, due to the changes in the social environment physical and mental development, and other factors of secondary school students, they may show problems such as rebelliousness and cognitive dissonance and other problems. The reason may be that Chinese parents are accustomed to excessive interference in their children’s lives, and do not give their children the right to make independent choices, which is prone to two extreme phenomena in the long run. One extreme phenomenon is that parents’ long-term interference in the growth of their children will make children unable to integrate external evaluation and recognition and their own ideas have been in a state of confusion, and they need to constantly seek external recognition to consolidate their sense of value, that is, pay too much attention to the evaluation of others, affected by external suggestions, resulting in children lacking happiness and goals, and living in full accordance with their parent’s expectations. It is easy to cause students to be influenced and manipulated by others, and then lack the ability of independent thinking and initiative. Another extreme phenomenon is that when parents exert various pressures and restrictions on children, children resist the social environment and expectations with amazing strength, and the rebellious behavior they show is to oppose the domination of parents, affirm their sovereignty, and even embark on the road of crime in an anti-social way.

There are significant gender differences in stress symptoms of secondary school students, which is generally consistent with the findings of previous studies (37, 38). The total score of stress symptoms and the scores of muscle tension and upper respiratory of girls were significantly higher than those of boys. The reason for this may be different from the way that boys and girls deal with stress. Boys have significantly higher levels of cortisol in their bodies when faced with stress or problems, and the compounds produced by cortisol are transported into the blood to act as energy, allowing the mind and will to be highly concentrated. However, when girls face stress or problems, the combination of oxytocin and estrogen in their bodies produces a “tend-and-befriend” response, opening a protective care mechanism that causes girls to escape behaviors or thoughts.

There are significant academic year differences in stress symptoms of secondary school students, which is basically consistent with the previous studies (39–41). The total score of stress symptoms and the scores of depression, sympathetic arousal, muscle tension, cognitive disorganization, and upper respiratory symptoms of senior high school students were significantly higher than those of junior high school students. The reasons may be as follows. First, the stress of entering a higher school leads senior high school students to the intensity of studying at school and reviewing at home. The lack of enough rest and entertainment makes senior high school students not have enough space to release pressure, and excessive accumulation of pressure is likely to cause psychological problems. Second, when senior high school students are in adolescence, the hormone levels in the body change significantly, and the sexual drive, agitation, aggression, and impulsivity also increase, which will lead to changes in the stress symptoms of senior high school students. Third, the physiological and mental conditions of senior high school students have basically reached the adult level, and they begin to care about their “adult role” and dignity. However, because there is still a significant gap between the life experience and minds of senior high school students and adults, they are often prone to anxiety and frustration in the face of their own experience and practical ability. In addition, the living conditions of the Chinese are generally higher, but many young people cannot find the motivation to struggle, feel confused about their future, and feel that their current life lacks meaning.

### 4.3 Analysis of the relationship between stress symptoms and stress events in secondary school students

There is a significant positive correlation between the scores and total scores of all dimensions of stress events and the scores and total scores of all dimensions of stress symptoms. Moreover, the scores and total scores of all dimensions of stress events of secondary school students grouped by gender and academic year are also significantly positively correlated with the scores and total scores of all dimensions of stress symptoms, which means the more stress events are, the more obvious stress symptoms of secondary school students are, which is basically consistent with existing research results (42). Stress events, as a stressor, are high-risk factors for stress symptoms in secondary school students, and stress events have a “cumulative effect.” Long-term experience of stress events will lead to the production of stress symptoms. This study also found that punishment and relationship stress had a higher impact on the stress symptoms of secondary school students than learning stress, and this result is inconsistent with the results of previous studies (41), which may be related to the improvement of individual self-esteem and the maintenance of good peer relationships among Chinese secondary school students. This study found that punishment, relationship stress, and learning stress were all important factors affecting stress symptoms in secondary school students, but the impact of punishment and relationship stress was higher than that of learning stress. This reason may be that as the Chinese people gradually become richer, the material support and care of parents for their children have improved the self-esteem of secondary school students. At the same time, the “double reduction” policy has extended the school time of secondary school students, which also makes students pay more attention to the maintenance of good peer relationships.

In terms of gender, the simple regression analysis of stress events on the stress symptoms of secondary school boys shows that the four dimensions of stress events, namely punishment, relationship, learning stress, and relationship stress, had significant positive predictive effects on each dimension of stress symptoms, and the adaption dimension had significant positive predictive effects on cognitive disorganization and upper respiratory symptoms. The influence of the punishment dimension on the stress symptoms of secondary school boys was the most significant. The simple regression analysis of stress events on the stress symptoms of secondary school girls shows that the loss dimension had no significant predictive effect on the symptoms of depression and muscle tension, and the other dimensions of stress events had a significant positive predictive effect on the symptoms of stress, among which the punishment dimension had the most significant effect on the symptoms of depression, nervous, cardiopulmonary and cognitive disorganization of secondary school girls. The relationship stress dimension had the most obvious effect on sympathetic arousal and muscle tension symptoms, and the learning stress dimension had the most obvious effect on upper respiratory symptoms. This indicates that although relationships, learning stress, punishment, loss, adaption, and other stress events are all risk factors for secondary school students’ stress symptoms, there are gender differences in the predictive effect of the same stress events on secondary school students’ stress symptoms (43). Compared with boys, in addition to the most obvious effect of punishment on depression, neurological, and cardiopulmonary arousal symptoms, relationship stress, adaptation, and learning stress events also had the most obvious effect on girls’ anger, sympathetic arousal, muscle tension, and upper respiratory. It is worth noting that since girls have higher empathy ability than boys and their overall relationships are better than boys (38, 40), the stress generated to maintain good relationships is more likely to be an important source of stress symptoms. Therefore, the mechanism of influence of stress events on stress symptoms is different between boys and girls in secondary school.

In terms of the academic year, the simple regression analysis of stress events on stress symptoms of junior high school students shows that the four dimensions of punishment, relationship, learning stress, and loss of stress events had significant positive predictive effects on all dimensions of stress symptoms of junior high school students, and the adaption dimension had significant positive predictive effects on anger, sympathetic arousal, cognitive disorganization, and upper respiratory symptoms. The influence of the punishment dimension on stress symptoms of junior high school students was most significant. Simple regression analysis of stress events on stress symptoms of high school students shows that each dimension of stress events had significant positive predictive effects on stress symptoms of senior high school students. The punishment dimension had the most significant impact on depression, anger, neurological, cardiopulmonary, and cognitive disorganization symptoms of senior high school students, and relationship stress had the most significant impact on sympathetic arousal, muscle tension, and upper respiratory symptoms. This indicates that there are academic year differences in the prediction effect of the same stress events in secondary school students, with the most obvious effect of relationship stress on the physiological symptoms of senior high school students, which is inconsistent with the results of previous studies (44). In junior high school, students mainly focus on family and school, and the relationship with their classmates is relatively simple, while in senior high school, students need to face more complex relationships and spend more time and energy maintaining the relationships. Therefore, the mechanisms of stress events on stress symptoms are also different between junior high school and senior high school students.

## 5 Conclusion

Through the horizontal investigation, it is found that the stress events and stress symptoms of Chinese secondary school students have significant differences in gender and academic year. According to the correlation and regression analysis of gender and academic year respectively, it is found that the same stress event has different influence mechanisms on the stress symptoms of Chinese secondary school students of different genders and different academic years. The influence of punishment on the stress symptoms of boys and junior high school students is the most significant, the influence of punishment, relationship stress, adaption, and learning stress on the stress symptoms of girls is the most significant, and the influence of punishment and relationship stress on the stress symptoms of senior high school students is the most significant. Limitations of the study include the use of a cross-sectional survey in this study, which can be verified by follow-up investigation or practical exploration in future studies, and the investigation of stress events and stress symptoms of Chinese secondary school students in this study, which can increase the investigation of primary school students and college students.

## Data availability statement

The raw data supporting the conclusions of this article will be made available by the authors, without undue reservation.

## Ethics statement

The studies involving humans were approved by the Ethics Committee of Nantong University. The studies were conducted in accordance with the local legislation and institutional requirements. Written informed consent for participation in this study was provided by the participants’ legal guardians/next of kin. Written informed consent was obtained from the minor(s)’ legal guardian/next of kin for the publication of any potentially identifiable images or data included in this article.

## Author contributions

AM: Conceptualization, Formal analysis, Funding acquisition, Investigation, Project administration, Writing – original draft, Writing – review & editing. ST: Conceptualization, Writing – review & editing, Investigation. JC: Data curation, Investigation, Writing – review & editing. HL: Conceptualization, Funding acquisition, Methodology, Project administration, Writing – review & editing.
